# Testosterone suppresses ventricular remodeling and improves left ventricular function in rats following myocardial infarction

**DOI:** 10.3892/etm.2015.2269

**Published:** 2015-02-06

**Authors:** XIAO-FEI WANG, XING-QIAN QU, TIAN-TIAN ZHANG, JUN-FENG ZHANG

**Affiliations:** 1 Department of Cardiology, Third People’s Hospital, Shanghai Jiao Tong University School of Medicine, Shanghai 201900, P.R. China; 2 Department of Anesthesiology, Third People’s Hospital, Shanghai Jiao Tong University School of Medicine, Shanghai 201900, P.R. China

**Keywords:** heart failure, testosterone, rats, atrial natriuretic peptide, brain natriuretic peptide, sarcoendoplasmic reticulum Ca^2+^-ATPase, matrix metalloproteinase, tissue inhibitor of metalloproteinase, glycogen synthase kinase, caspase-3, cardiomyocyte apoptosis, ventricular remodeling

## Abstract

Men with congestive heart failure (CHF) have relatively low testosterone levels. Several studies demonstrated that testosterone treatment increases cardiac output and reduces peripheral vascular resistance. However, the effects of testosterone on heart function, cardiomyocyte apoptosis and ventricular remodeling have not been fully elucidated. This study was conducted to investigate the effects of testosterone on heart function, cardiomyocyte apoptosis and ventricular remodeling in male rats post-myocardial infarction. A total of 86 male rats were randomly assigned to undergo ligation of the coronary artery (n=70) or pseudosurgery (n=16). After 6 weeks, a left ventricular ejection fraction (LVEF) of ≤45% was defined as a successful model of CHF. The model rats were randomly assigned to 3 groups, namely low-dose testosterone (TU), high-dose TU and placebo (PL) groups. After treatment for 12 weeks, the expression of several mRNA transcripts in myocardial tissue was measured by quantitative polymerase chain reaction. Immunofluorescence was used to measure myocardial caspase-3 expression. Compared to the PL group, LVEF was significantly improved in the TU treatment groups. Moreover, the mRNA expression of atrial natriuretic peptide, brain natriuretic peptide, matrix metalloproteinase-2 and sarcoendoplasmic reticulum Ca^2+^-ATPase 2a was significantly reduced, while the mRNA expression of glycogen synthase kinase 3β and tissue inhibitor of metalloproteinase-2 was markedly increased in the TU groups. TU treatment also significantly reduced caspase-3 expression. Therefore, different doses of TU suppressed ventricular remodeling and improved left ventricular function, reduced apoptosis and prevented mortality in a CHF rat model.

## Introduction

Chronic heart failure (CHF) is a leading cause of morbidity and mortality worldwide (1). The currently used pharmacological treatments of CHF include diuretics, vasodilators, inotropic agents and neurohormonal manipulation, such as angiotensin-converting enzyme inhibitors, β-blockers and aldosterone receptor antagonists. These drugs improve the clinical symptoms of CHF, elevate the left ventricular ejection fraction (LVEF) and may reduce mortality and hospitalization time. However, even with optimal pharmacological therapy, the annual mortality rate of patients with CHF remains high (1).

Previous studies investigated the cellular factors and ventricular remodeling mechanisms involved in the pathogenesis of CHF and found that the expression of atrial natriuretic peptide (ANP) and brain natriuretic peptide (BNP) increased significantly, while the expression or activity of negative regulators of cardiac hypertrophy signaling, i.e., glycogen synthase kinase-3β (GSK-3β) and sarcoendoplasmic reticulum Ca^2+^-ATPase 2a (SERCA2a) decreased in CHF (2–3); this imbalance may affect left ventricular systolic function, leading to cardiac enlargement, myocardial apoptosis, skeletal muscle atrophy and cardiac cachexia (1) and is one of the important activators of matrix metalloproteinases (MMPs). MMPs are involved in the proteolytic degradation of the extracellular matrix and play an important role in ventricular remodeling (4).

Men with CHF have been shown to have low testosterone (TU) levels (5). Several animal studies and small clinical trials demonstrated that TU supplementation may increase cardiac output and reduce peripheral vascular resistance (6–9). However, as the effects of TU on myocardial characteristics have not been fully elucidated, the patients often have reservations regarding TU treatment. Thus, we conducted the present study to investigate the effects of TU on mortality and heart function improvement in rats surviving from acute myocardial infarction. The expressions of ANP, BNP, GSK-3β, SERCA2a, MMP-2, tissue inhibitor of MMP-2 (TIMP-2) and caspase-3 in myocardial tissue were also investigated.

## Materials and methods

### Animals

This study was approved by the Institutional Review Board of Shanghai Second Military Medical University. The investigation conformed with the Guide for the Care and Use of Laboratory Animals published by the US National Institutes of Health (NIH Publication no. 85–23, revised 1996). A total of 86 Sprague-Dawley male rats (8 weeks old and weighing 270±20 g) were purchased from the Experimental Animal Center of Shanghai Second Military Medical University. The rats were housed in an air-conditioned room with a 12:12 h dark-light cycle and were allowed free access to standard feed and tap water.

### Surgical procedures

The following surgical procedures were conducted at the Laboratory of Shanghai Second Military Medical University (Shanghai, China). Myocardial infarction was induced in 70 rats. Under general anesthesia (i.p. pentobarbital 40 mg/kg), the rats were intubated and ventilated with oxygen using a positive-pressure respirator. The respiratory parameters were as follows: tidal volume, 7–9 ml; respiratory rate, 60 breaths/min; and breathing ratio, 1:2. A left thoracotomy was performed at the level of the fourth intercostal space, the lungs were retracted to expose the heart and the pericardium was opened. The proximal left anterior descending coronary artery was ligated between the pulmonary outflow tract and the edge of the left atrium, with a 6-0 atraumatic suture. The needle depth was ~0.5 mm. Acute myocardial ischemia was fully developed when the anterior wall of the left ventricle turned pale, with concurrent ST segment elevation on the electrocardiogram (10). The lungs were then inflated by increasing the positive end-expiratory pressure and the incision was closed, followed by recovery of the intrathoracic negative pressure, ventilator weaning and awakening the rats from anesthesia. Penicillin (800,000 U i.m.) was continuously used to prevent infection in the 3 days following surgery. Pseudosurgery (PS) was performed on another 16 rats by passing a silk suture under the coronary artery, but not ligating it. At 6 weeks after the surgery, the 60 surviving rats underwent ultrasound examination.

Of the 86 rats in which myocardial infarction was induced, 60 rats (operative mortality rate, 63.83%) with a LVEF of ≤45% were assigned to the CHF model group (10). The 60 rats were then randomized into the low-dose TU, the high-dose TU and the placebo (PL) groups (n=20 per group), which received 5 mg/kg TU i.m. (Xianju Pharmaceutical Inc., Zhejiang, China), 500 mg/kg TU i.m. and 5 mg/kg sterile peanut oil i.m., respectively, every 2 weeks for a total of 12 weeks following surgery. The rats in the PS group (n=16) were treated with an equal amount of sterile peanut oil for 12 weeks. At 18 weeks after surgery, the chest was opened and the heart was arrested at diastole by intraventricular injection of 15% KCl. After the heart was weighed, the left ventricle was sectioned transversely into 3 slices, rapidly frozen in isopentane and stored at −70°C for tissue analysis.

### TU supplementation dose estimation methods

Based on the established dosing guidelines for TU (http://drugs.dxy.cn/drug/55434.htm), castrated male rats were injected with 13.7 mg/kg to maintain normal androgen action. The half-life of TU is 15 days; thus, castrated rats were supplemented at a dose of ~13.7 mg/kg every 2 weeks. Furthermore, CHF patients exhibit a decrease in serum testosterone of ~30–60% (11). Thus, based on projections of this reduction, we performed preliminary experiments using 9, 6.8, and 5 mg/kg every 2 weeks. At 6 weeks after treatment, the serum testosterone concentrations were measured. We found that treatment with 5 mg/kg TU every 2 weeks resulted in TU concentrations more similar to those of the PS group; thus, 5 mg/kg every 2 weeks was selected as the low-dose group. The high-dose group was treated with 500 mg/kg TU every 2 weeks (12).

### Echocardiography

Transthoracic echocardiography was performed on the 6th and 18th weeks following surgery using a Vivid-7 Ultrasound system (GE Healthcare Bio-Sciences, Pittsburgh, PA, USA) with an 11.4-MHz phased array transducer under anesthesia and mechanical ventilation as previously described. When the rats were anesthetized, M-mode echocardiograms, guided by 2-dimensional long-axis images, were obtained through the anterior and posterior left ventricular walls at the level of the papillary muscles. The left ventricular end-systolic diameter (LVEDs) and the left ventricular end-diastolic diameter (LVEDd) was measured from the M-mode tracing using the leading-edge method of the American Society for Echocardiology. The LVEF was determined using the formula: LVEF = (LVEVd − LVEVs)/LVEVd × 100% (10). An LVEF of ≤45% was defined as heart failure.

The echocardiographer who performed the assessment of the left ventricular function was blinded to the animal grouping. Intra-observer variations in LVEDs, LVEDd and LVEF were <4%.

### Radioimmunoassay of serum testosterone level

Serum testosterone levels were measured preoperatively and at 6 and 18 weeks following surgery, using an anti-testosterone antibody (clone 3T16; RIA box; Kemei Dongya Institute of Biotechnology, Beijing, China) according to the manufacturer’s instructions. The intra- and inter-assay coefficients of variation were 7.4 and 9.5%, respectively.

### Left ventricular mass index (LVMI)

Following removal of the heart, the left ventricle (including the interventricular septum) was weighed. The ratio of left ventricular mass and body weight as the left ventricular mass index (LVMI).

### Quantitative polymerase chain reaction (qPCR)

qPCR was used to determine the mRNA expression of ANP, BNP, SERCA2a, MMP-2, TIMP and GSK-3β in myocardial tissues. Approximately 20–30 mg of myocardial tissue was placed in a 1.5-ml microfuge tube. TRIzol reagent (1 ml; Invitrogen Life Technologies, Carlsbad, CA, USA) was added and the tissues were homogenized to extract RNA. A Reverse Transcription kit (Takara Biotechnology, Dalian, China) was used to transcribe 2 μg total RNA into single-stranded cDNA. The mixture contained 8.0 μl 5X PrimeScript Buffer, 2.0 μl PrimeScript RT Enzyme Mix, 2.0 μl 100 μM random primers, 2.0 μl of 50 μM Oligo dT primer, 2.0 μg of RNA (dissolved in RNase-free water) and RNase-free water up to a final volume of 40 μl. The mixture was incubated at 37°C for 15 min and at 85°C for 5 sec. The obtained cDNA was stored at −20°C until use. Sample preparation, PCR pipetting and PCR product detection analyses were completed in separate work spaces. Vecter NTI 11.0 software (Vector Software, East Greenwich, RI, USA) was used to design forward and reverse primers for the specific targets (Table I).

qPCR was performed on a Roche LightCycler 480 instrument (Roche Diagnostics, Basel, Switzerland). A melting curve analysis was performed to assess the specificity of the primer sets for the target gene.

### Immunofluorescence staining

Determination of the expression of caspase-3 in the myocardium was conducted as follows: The paraffin sections were heated at 65°C for 2 h. The sections were then dewaxed in water and washed 3 times with PBS (pH 7.4) for 5 min per wash. The sections were placed in EDTA buffer and boiled for 10 min. After cooling at room temperature, the sections were washed 3 times for 5 min per wash in PBS (pH 7.4). After drying, the sections were incubated with 5% bovine serum albumin for 20 min, followed by incubation with ~50 μl of rabbit anti-mouse caspase-3 antibody (BA2142; dilution, 1:100; Wuhan Boster Biological Technology Co., Ltd., Wuhan, China) at 4°C overnight. Following incubation, the samples were washed with PBS 3 times for 5 min per wash. Finally, the sections were incubated with 50–100 μl CY3-labeled goat anti-rabbit fluorescent secondary antibodies (111-165-003; Jackson ImmunoResearch Laboratories, Inc., West Grove, PA, USA) in the dark at room temperature for 50 min to 1 h. Following washing with PBS, each section was incubated with 50–100 μl DAPI nuclear staining for 5 min in the dark. The sections were slightly dried and then mounted and sealed with the anti-fluorescence quenching reagent (Fluorescent Mounting Media; KPL, Gaithersburg, MD, USA) at 4°C in the dark.

Image-Pro Plus 6.0 software was used to analyze immunofluorescence signals from images captured with an inverted Nikon fluorescence microscope (80i; Nikon Corporation, Tokyo, Japan). Caspase-3 expression was detected using an excitation wavelength of 510–560 nm and the corresponding DAPI signal was detected using an excitation wavelength of 330–380 nm. Between 3 and 5 random visual fields were selected for each section (magnification, ×200). Image-Pro Plus 6.0 software was used to convert the fluorescence images to black and white images and the integrated optical density (IOD value) of each image was determined. The larger the IOD value, the stronger the positive expression. Values are reported as mean ± SD IOD value for each group.

### Statistical methods

All the experimental data are expressed as means ± SD and were processed with SPSS 17.0 software (SPSS Inc., Chicago, IL, USA). Comparisons of data between groups were performed using analysis of variance. Chi-square tests were used to compare mortality rates between the TU and PL groups. Pairwise comparisons were performed using the least significant difference t-test. P<0.05 was considered to indicate a statistically significant difference.

## Results

### TU suppresses ventricular remodeling

#### TU decreases the expression of ANP and BNP mRNA

**Figure 1:**
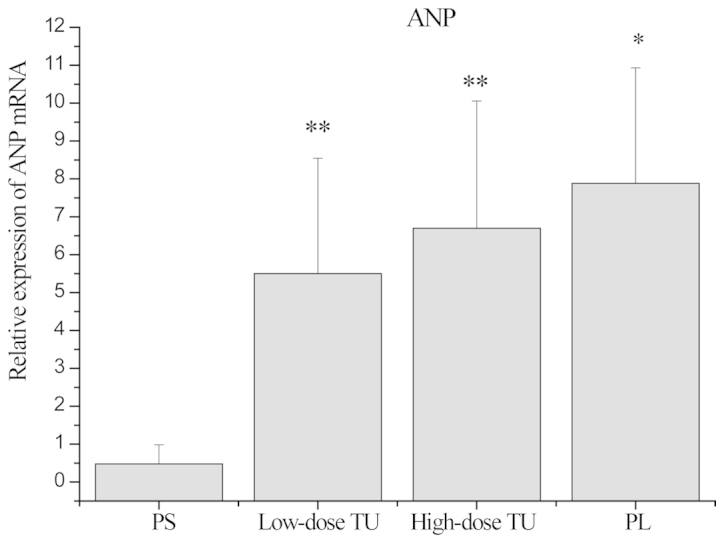
Relative expression of atrial natriuretic peptide (ANP) mRNA in myocardial tissue in each group as determined by reverse transcription-quantitative polymerase chain reaction. ^*^P<0.05, compared to the PS group; ^**^P<0.05, compared to the PL group. PS, pseudosurgery; TU, testosterone; PL, placebo.

**Figure 2:**
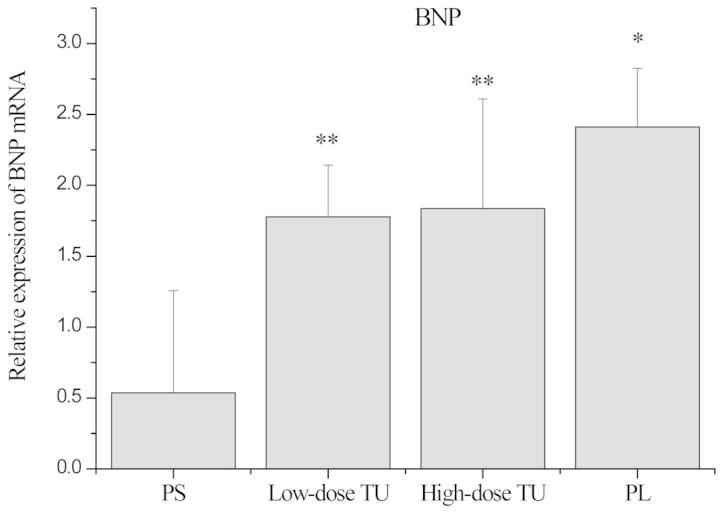
Relative expression of brain natriuretic peptide (BNP) mRNA in myocardial tissue in each group as determined by reverse transcription-quantitative polymerase chain reaction. ^*^P<0.05, compared to the PS group; ^**^P<0.05, compared to the PL group. PS, pseudosurgery; TU, testosterone; PL, placebo.

The expression of ANP and BNP mRNA in myocardial tissue was significantly higher in the PL group compared to that in the PS group (P<0.05). However, compared to the PL group, the TU groups exhibited lower myocardial expression of ANP and BNP mRNA (P<0 05). There was no significant difference in the expression of these genes between the low- and high-dose TU groups (Figs. 1 and 2).

#### TU decreases the expression of GSK-3β mRNA and increases the expression of SERCA2a mRNA

**Figure 3:**
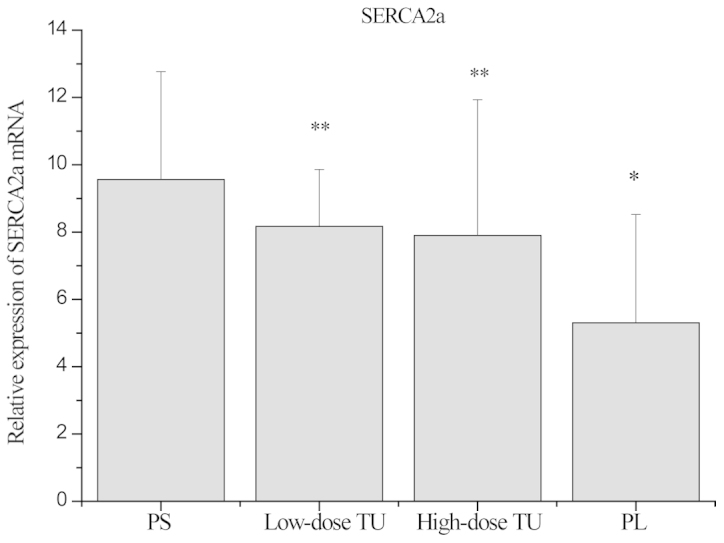
Relative expression of sarcoendoplasmic reticulum Ca^2+^-ATPase 2a (SERCA2a) mRNA in myocardial tissue in each group as determined by reverse transcription-quantitative polymerase chain reaction. ^*^P<0.05, compared to the PS group; ^**^P<0.05, compared to the PL group. PS, pseudosurgery; TU, testosterone; PL, placebo.

**Figure 4:**
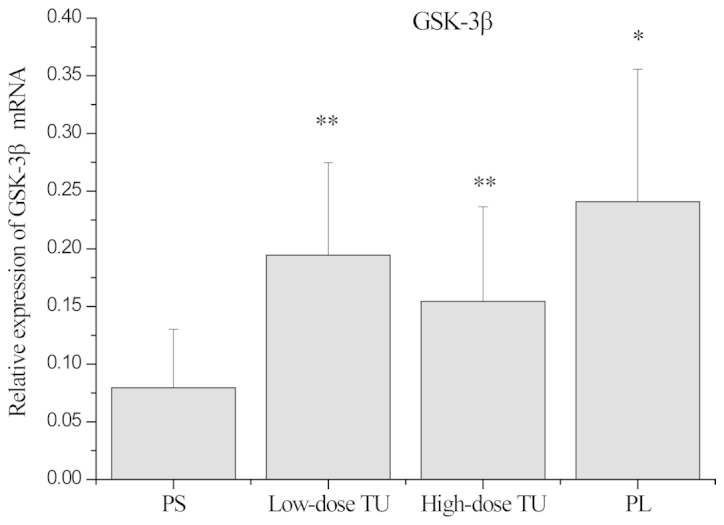
Relative expression of glycogen synthase kinase-3β (GSK-3β) mRNA in myocardial tissue in each group as determined by reverse transcription-quantitative polymerase chain reaction. ^*^P<0.05, compared to the PS group; ^**^P<0.05, compared to the PL group. PS, pseudosurgery; TU, testosterone; PL, placebo.

The expression of GSK-3β mRNA was significantly higher in the PL group compared to that in the PS group. However, TU treatment led to a significant reduction in the myocardial expression of GSK-3β mRNA. The expression of SERCA2a mRNA was decreased significantly in the PL group compared to that in the PS group, and TU treatment led to a significant increase in SERCA2a mRNA expression (P<0.05). There was no significant difference in the expression of either GSK-3β or SERCA2a mRNA between the low- and high-dose TU groups (Figs. 3 and 4).

#### TU decreases the expression of MMP-2 transcripts and increases the expression of TIMP transcripts

**Figure 5:**
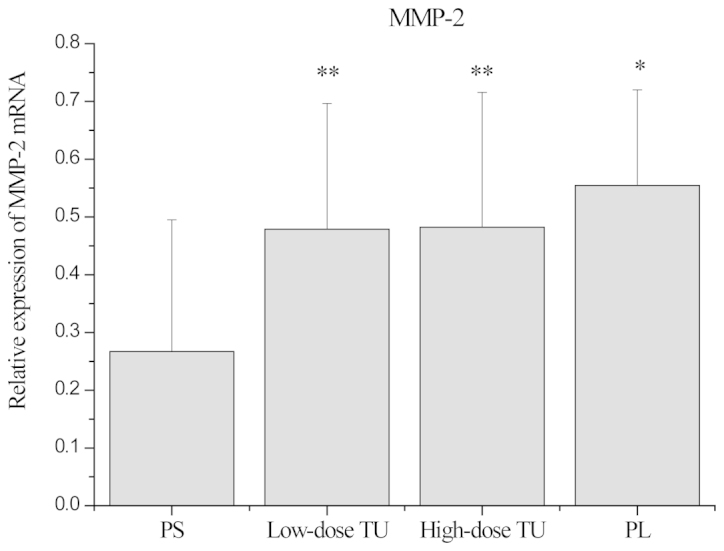
Relative expression of matrix metalloproteinase-2 (MMP-2) mRNA in myocardial tissue in each group as determined by reverse transcription-quantitative polymerase chain reaction. ^*^P<0.05, compared with the PS group; ^**^P<0.05, compared with the PL group. PS, pseudosurgery; TU, testosterone; PL, placebo.

**Figure 6:**
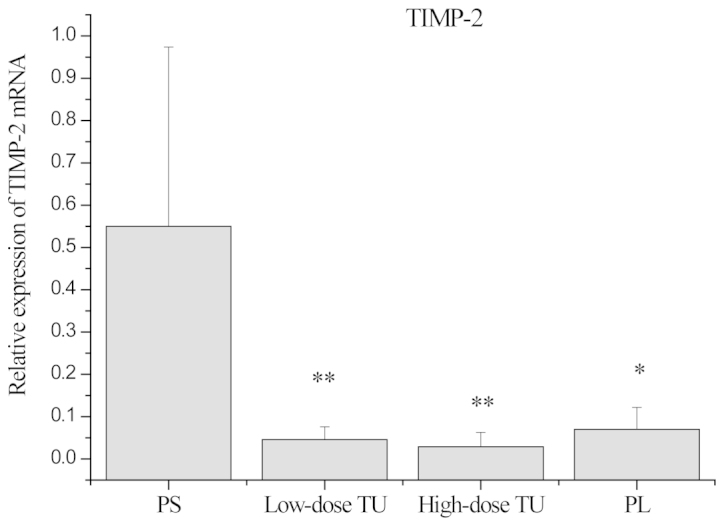
Relative expression of tissue inhibitor of metalloproteinase-2 (TIMP-2) mRNA in myocardial tissue in each group as determined by reverse transcription-quantitative polymerase chain reaction. ^*^P<0.05, compared with the PS group; ^**^P<0.05, compared with the PL group. PS, pseudosurgery; TU, testosterone; PL, placebo.

There was a 2-fold decrease in MMP-2 mRNA level in the PL group compared to the PS group (P<0.05). TU treatment led to a 20% decrease in the expression of MMP-2 mRNA compared to the PL group. TIMP mRNA expression was lower in the PL group compared to that in the PS group, but was markedly increased in the TU groups compared to the PL group (P<0.05). There was no significant difference in either MMP-2 or TIMP-2 mRNA level between the high- and low-dose TU groups (Figs. 5 and 6).

#### TU may reduce apoptosis by decreasing the expression of caspase-3

**Figure 7:**
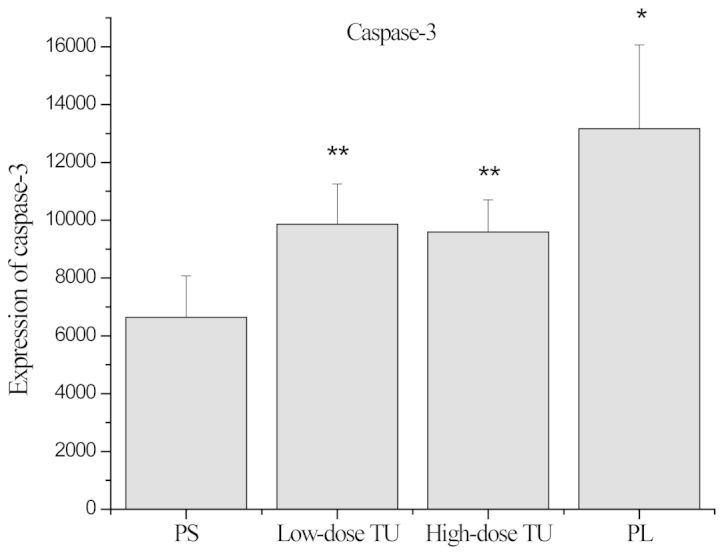
Expression of caspase-3 in myocardial tissue in each group as determined by immunofluorescence. ^*^P<0.05, compared to the PS group; ^**^P<0.05, compared to the PL group. PS, pseudosurgery; TU, testosterone; PL, placebo.

**Figure 8:**
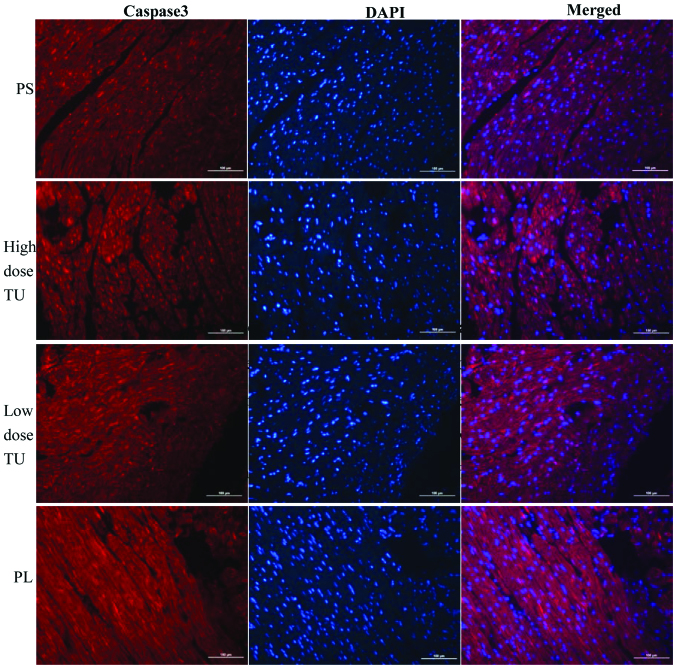
Expression of caspase-3 in each group. Bar, 100 μm. Sample size, n=5. Caspase-3 was mainly expressed in the myocardial cell cytoplasm and nucleus. The fluorescent expression of caspase-3 in the PL group was higher than that in the PS group (P<0.05). However, treatment with TU led to a significant decrease in caspase-3 expression (P<0.05). TU, testosterone; PS, pseudosurgery; PL, placebo.

The expression of caspase-3 in the PL group was significantly higher compared to that in the PS group (P<0.05). However, TU treatment led to a significant decrease in caspase-3 expression (P<0.05). There was no difference in caspase-3 expression between the low- and high-dose TU groups (Figs. 7 and 8).

#### TU improves left ventricular function and decreases mortality

**Figure 9:**
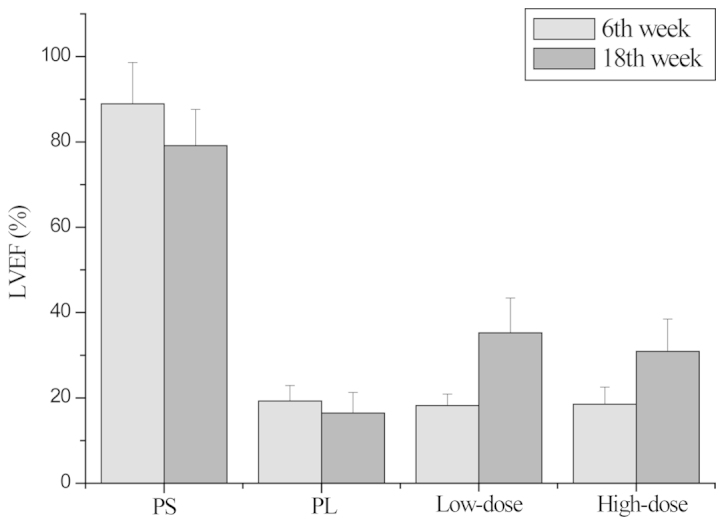
Left ventricular ejection fraction (LVEF) in each group. There was a significant reduction in the LVEF in the PL and TU groups at 6 weeks (P<0.05). At 18 weeks, the LVEF in the PL group remained low, but had improved in the TU group (P<0.05). There was no significant difference in LVEF between the low- and high-dose TU groups (P>0.05) TU, testosterone; PS, pseudosurgery; PL, placebo.

There was a significant reduction in the LVEF in the PL and TU groups at 6 weeks following surgery, compared to that in the PS group (Table II and III, and Fig. 9, P<0.05). At the end of the study, the LVEF in the PL group remained low, but it had improved in the TU group (Table II and III, and Fig. 9; P<0.05).

**Figure 10:**
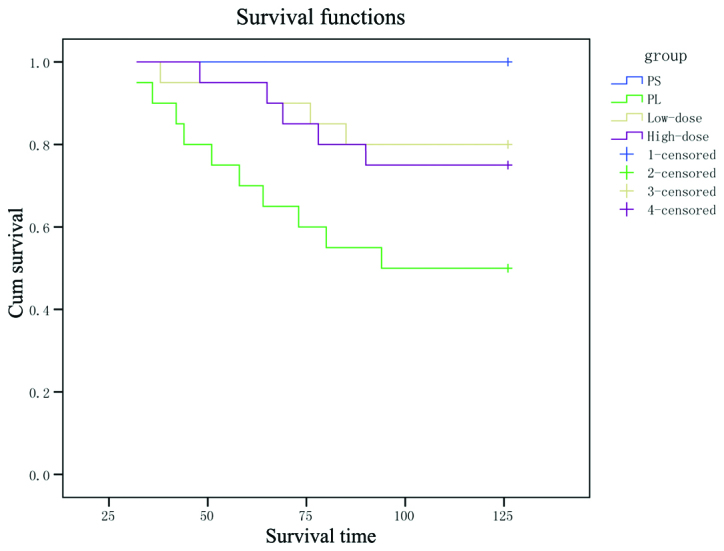
Kaplan-Meier survival functions curve in each group. PS, pseudosurgery; PL, placebo; Cum, cumulative.

At the end of the study, the mortality rate in the TU group was lower compared to that in the PL group (Table II; P<0.05). There was no difference in mortality between the low- and high-dose TU groups (Table II; P>0.05). Kaplan-Meier survival curves were constructed for each group (Fig. 10)

#### TU causes an increase in body weight

**Figure 11:**
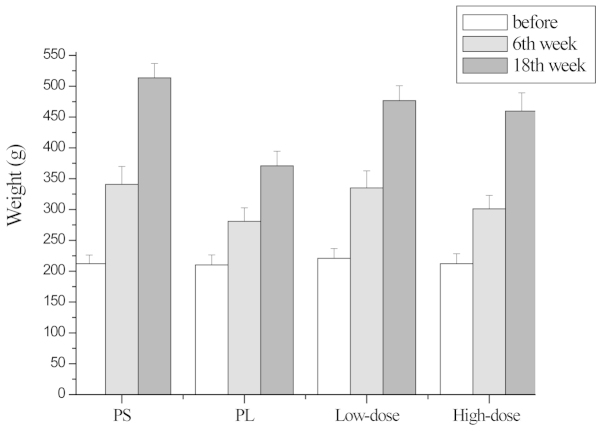
Body weight changes in each group. The PL group was lower than that the PS group at both 6 and 18 weeks. The TU group was greater than that in the PL group at 18 weeks after the surgery (P<0.05). There was no significant difference in the low- and high-dose TU groups (P>0.05). TU, testosterone; PS, pseudosurgery; PL, placebo.

In all the groups, there was an increase in body weight during the study. However, the mean body weight of the PL group was lower compared to that in the PS group at 6 and 18 weeks after surgery. The mean body weight in the TU group was higher compared to that in the PL group at 18 weeks after surgery (Table II and Fig. 11, P<0.05). There was no significant difference in body weight between the low- and high-dose TU groups (Table II and Fig. 11, P>0.05).

#### Serum TU levels

**Figure 12:**
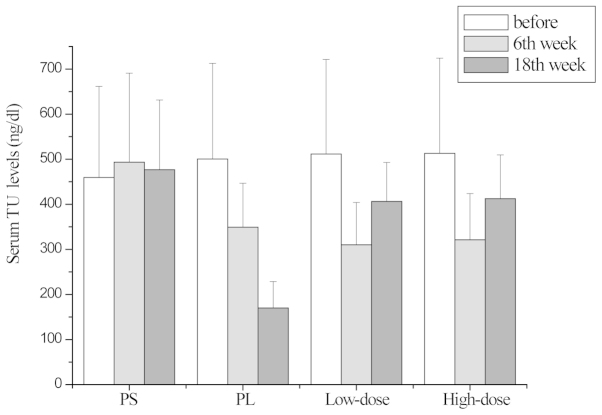
Serum TU level in each group. The average TU levels in the PL group 6th and 18th weeks was lower than the PS (P<0.05). In the PL group, the mean TU level at 18th weeks was lower than the 6th week (P<0.05). In the TU group, the mean TU level at 18th weeks was higher than at 6th week, and was higher than the PL group (P<0.05). There was no difference between the low- and high-dose TU groups (P>0.05). TU, testosterone; PS, pseudosurgery; PL, placebo.

The average TU levels in the PL group at 6 and 18 weeks after surgery were significantly lower compared to those in the PS group (Table II and Fig. 12, P<0.05). In the PL group, the mean serum testosterone level at 18 weeks after surgery were lower compared to those at 6 weeks (Table II and Fig. 12, P<0.05). In the TU groups, the mean TU level at 18 weeks was higher compared to that at 6 weeks and it was higher compared to that in the PL group at the corresponding time point (Table II and Fig. 12, P<0.05). There was no difference in mortality between the low- and high-dose TU groups (Table II and Fig. 12, P<0.05).

## Discussion

In this study, we observed a significant increase in the body weight of PS rats at 18 weeks after surgery. By contrast, the body weights of PL rats at 18 weeks were significantly lower compared to those in the PS group, suggesting the presence of cardiogenic cachexia. Despite the fact that body weights in the TU group at 6 and 18 weeks after surgery were significantly increased compared to those in the PL group, the rats receiving TU still exhibited lower body weight compared to those in the PS group. Prompt improvement of the cachexic status may be part of the mechanism underlying TU-dependent improvement in CHF, i.e., TU-mediated improvement in heart function may lead to increased body weight. However, the neuroendocrine metabolic imbalance observed in CHF is caused by a number of abnormal systemic and molecular events and, therefore, TU replacement therapy may only correct metabolic disorders in CHF to a certain extent.

In CHF, MMP expression increases, allowing degradation of the normal components of the extracellular matrix. The increased expression of MMPs may also lead to the formation of abnormal structures and the dysfunction of collagen and connective tissue, eventually destroying the heart fibrillar collagen network and promoting left ventricular fibrosis and expansion, thereby permitting ventricular remodeling. MMP-2 belongs to the MMP family of gelatin enzymes and its expression was significantly increased following CHF surgery in rats, but was markedly decreased following treatment with TU. Therefore, TU treatment with may improve myocardial fibrosis of patients with CHF.

The expression of ANP and BNP mRNA is very low in normal myocardial tissue after birth. These genes belong to the group of embryonic genes and act as pathological cardiac hypertrophy reliability indicators (15). It was previously demonstrated that the expression of ANP and BNP may increase up to 10-fold in the ventricular muscle of the heart or in cultured cardiomyocytes treated with growth factors that stimulate cardiac hypertrophy (6,7). In the present study, we observed that ANP and BNP mRNA levels were significantly higher in rats that underwent surgery to mimic CHF. Moreover, TU treatment reduced the expression of these genes. Therefore, TU treatment may improve the prognosis of patients with CHF.

GSK-3β is important negative regulator of cardiac hypertrophy and plays an important role in the regulation of myocardial apoptosis (16). Overexpression of GSK-3β may prevent the development of cardiac hypertrophy. SERCA2a is an isoform of the SERCA family of proteins and reduced expression or activity of this protein has been reported in CHF (2,3). SERCA plays a major role in the regulation of intracellular Ca^2+^ and directly affects myocardial function. Increase or decrease in SERCA activity may lead to heart failure and arrhythmia (17). Moreover, the reduced activity or expression of SERCA2a may lead to myocardial cell dysfunction (3). Our preliminary results demonstrated that low-dose TU increased the mRNA expression of GSK-3β and SERCA2a, possibly affecting myocardial hypertrophy.

Our study demonstrated that TU supplementation regulated the abovementioned agents and reduced the heart weight, indicating a role for TU therapy in the treatment of CHF. Our study also demonstrated that the ameliorative effects of TU supplementation on CHF are dose-independent, as low-dose TU was sufficiently efficacious. Therefore, it may be concluded that TU inhibits ventricular remodeling and prevents ventricular hypertrophy.

Cardiomyocyte apoptosis is an important mechanism involved in heart failure. The interleukin-1β-converting enzyme gene, which encodes a cysteine aspartate-specific protease-caspase, plays a central role in the apoptotic process (18). It was previously reported that, in patients with ischemia/reperfusion treated with caspase inhibitors early after the event, there was a reduction in myocardial infarct size, left ventricular end-diastolic pressure and apoptosis of myocardial cells (19,20). Our study demonstrated that TU treatment reduced myocardial apoptosis, as evidenced by caspase-3 expression, thereby providing insights into the basis for TU treatment in CHF.

Based on our findings, we were able to conclude the following: i) Following myocardial infarction, the LVEF in male rats was significantly decreased; ii) low-dose TU reduced the mRNA expression of MMP-2, ANP and BNP and increased the mRNA expressions of GSK-3β and SERCA-2a in myocardial tissue, thereby improving immune imbalance and ventricular remodeling; iii) low-dose TU reduced the expression of caspase-3, a protein critical to apoptosis, thus suppressing myocardial apoptosis; iv) low-dose TU following myocardial infarction improved heart function in male rats; and v) low-dose TU reduced mortality following myocardial infarction in male rats. These results may have implications for the use of TU in the treatment of CHF.

## Figures and Tables

**Table I:** Primer sequences for the amplified genes.

Gene name	Forward (5′-3′)	Reverse (3′-5′)	Length (bp)
SERCA2a	TCTGACTTTCGTTGGCTGTG	GCCTTTGTTATCCCCAGTGA	121
ANP	ATCTGATGGATTTCAAGAACC	CTCTGAGACGGGTTGACTTC	169
BNP	ACAATCCACGATGCAGAAGCT	GGGCCTTGGTCCTTTGAGA	91
MMP-2	CTGGGCAACAAGTATGAGAG	GTGTAGGTGTAGATAGGGGC	218
TIMP-2	GCATCACCCAGAAGAAGAGC	GTTTCCAGGAAGGGATGTCA	338
GSK-3β	ATGGCAGCAAGGTAACCACAG	TCTCGGTTCTTAAATCGCTTGTC	193
GAPDH (reference gene)	TGCTGAGTATGTCGTGGAGT	GTCTTCTGAGTGGCAGTGAT	288

SERCA2a, sarcoendoplasmic reticulum Ca^2+^-ATPase 2a; ANP, atrial natriuretic peptide; BNP, brain natriuretic peptide; MMP-2, matrix metalloproteinase-2; TIMP-2, tissue inhibitor of MMP-2; GSK-3β, glycogen synthase kinase-3β; GAPDH, glyceraldehyde 3-phosphate dehydrogenase.

**Table II:** Mortality rate, body weight changes and left ventricular ejection fraction (LVEF) percentages in the different groups.

	PS	PL	TU	
			
Parameters	(n=16)	(n=20)	Low-dose (n=20)	High-dose (n=20)
Total no. of rats	16	20	20	20
Deaths	0	10	4	5
Mortality rate (%)	0	50^a^	20	25^d^
Body weight (g)				
Before surgery	212.13±14.01	210.22±16.08	220.84±15.91	212.19±16.14
At 6 weeks	340.72±28.94	280.77±22.07^a^	334.98±27.74^b^	301.12±21.74^b^,^d^
At 18 weeks	513.56±23.17^c^	370.78±23.80^a^,^c^	476.65±24.11^b^,^c^	459.76±29.47^b^,^c^,^d^
LVEF %				
At 6 weeks	88.9±9.7	19.3±3.6^a^	18.21±2.7^b^	18.53±4.0^b^,^c^,^d^
At 18 weeks	79.12±8.5	16.47±4.8^a^,^c^	35.25±8.2^b^,^c^	30.89±7.6^c^,^d^
Serum TU levels (ng/dl)				
Before surgery	459.6±201.7	500.4±212.3	511.6±209.5	512.9±211.3
At 6 weeks	493.5±197.4	349.2±97.4^a^	310.2±93.8^b^	321.3±102.6^b^,^d^
At 18 weeks	476.8±154.7	170.1±58.8^a^,^c^	406.5±86.2^b^,^c^	412.5±97.1^c^,^b^,^d^

PS, pseudosurgery; PL, placebo; TU, testosterone.

a PL group compared to PS group, P<0.05.

b TU groups compared to PL group, P<0.05.

c Comparison of 6th to 18th week within the same group, P<0.05.

d Low-dose TU group compared to high-dose TU group, P>0.05.

Values are presented as means ± standard deviation.

**Table III:** Ultrasound data in each group.

					TU			
			
	PS (n=16)		PL (n=20)		Low-dose (n=20)		High-dose (n=20)	
				
Parameters	6 weeks	18 weeks	6 weeks	18 weeks	6 weeks	18 weeks	6 weeks	18 weeks
IVST	1.47±0.25	1.58±0.24	1.43±0.18	1.58±0.18	1.50±0.39	1.69±0.19	1.51±0.22	1.67±0.20
PWT	1.58±0.27	1.67±0.31	1.60±0.20	1.76±0.28	1.60±0.21	1.77±0.18	1.62±0.21	1.78±0.26
LVEVs	0.12±0.03	0.11±0.04	0.63±0.21	0.67±0.22	0.63±0.20	0.45±0.21	0.61±0.25	0.47±0.18
LVEVd	0.34±0.07	0.36±0.9	0.96±0.20	0.94±0.20	0.93±0.19	0.76±0.23	0.92±0.17	0.79±0.19
LVEF%	88.9±9.7	79.12±8.5	19.3± 3.6^a^	16.47±4.8^a^,^c^	18.21±2.7^b^	35.25±8.2^b^,^c^	18.53±4.0^b^,^c^,^d^	30.89± 7.6^c^,^d^

TU, testosterone; PS, pseudosurgery; PL, placebo; IVST, interventricular septal thickness; PWT, posterior wall thickness; LVEF, left ventricular ejection fraction; LVEVs, left ventricular end-systolic volume; LVEVd, left ventricular end-diastolic volume.

a PL group compared to PS group, P<0.05.

b TU group compared to PL group, P<0.05.

c Comparison at 6 and 18 weeks within the same group, P<0.05.

d Low-dose TU group compared to high-dose TU group, P>0.05.
